# Supplementary material for: Nonvolatile Ternary Memristor Based on Fluorene-Benzimidazole Copolymer/Au NP Composites
Source: Nanomaterials (Basel). 2022 Nov 22;12(23):4117. doi: 10.3390/nano12234117 (PMC9741367; doi:10.3390/nano12234117)
Supplement: Supplementary file 1 [file nanomaterials-12-04117-s001.zip › nanomaterials-2020562-supplementary.pdf]

Supporting Information

Nonvolatile Ternary Memristor Based on Fluorene-Benzimidazole Copolymer/Au NP Composites

Meng Gao, Yanting Du, Haifeng Yu, Zhaohua He, Shuhong Wang* and Cheng Wang*

Contents

1. Preparation of M-BBO, PF-BBO and Au NPs...………………3
2. ^{1}H NMR and ^{13}C NMR spectra of M-BBO……...…………………4
3. ^{1}H NMR spectrum of PF-BBO……………………….…………5
4. FT-IR spectrum of PF-BBO...…………...………………………6
5. Molecular weight and dispersion of PF-BBO………………...7
6. Optical and electrochemical properties….…………………...7

1. Preparation of M-BBO, PF-BBO and Au NPs

Preparation of M-BBO: 3,6-dibromo-1,2-phenylenediamine and 1,8-naphthalene dicarboxylic anhydride were thoroughly ground and added to a Schlenk bottle containing acetic acid. Under the protection of nitrogen, the mixture was heated to 110 °C and whipped at a constant temperature for 8 h. The liquid after the reaction was poured into deionized water, and the products were extracted under vacuum and dried for 12 h. Ultimately, column chromatography was applied to refine the preceding substances into a yellow solid. The monomer was named 9,12-dibromo-7H-benzimidazo-[2,1-a]benzo[de]isoquinolin-7-one (M-BBO).

Preparation of PF-BBO: The M-BBO, 9,9-dioctylfluoren-2,7-diborane bis (1,3-propylene glycol) ester, K_2CO_3 solution, tetra (triphenylphosphine) palladium catalyst, and dehydrated toluene were added into the Schlenk flask. The reaction was conducted for 48 h at 108 °C with constant temperature magnetic stirring in nitrogen. After washing with deionized water until the aqueous phase is clarified, the polymer layer is dropped into a rapidly stirred beaker containing iced methanol. The precipitated polymer was collected by vacuum extraction and filtration method. After vacuum drying, the polymeric compound was refined more precisely by the Soxhlet extraction method. After vacuum drying, the final orange

product was obtained. The polymer was named poly[2,7-(9,9-dioctyl)-fluorene-*alt*-7H-benzimidazo-[2,1-a]benzo[de]isoquinolin-7-one] (PF-BBO).

Preparation of Au NPs: First, solutions of 0.1% $HAuCl_4$ and 2% sodium citrate were prepared, then a small amount of $HAuCl_4$ solution is diluted to 200 mL with deionized water, and heated, stirred until boiling; the sodium citrate solution is added and heated until wine red, then heating is stopped and stirring continues until room temperature is reached. The resulting Au NPs solution was a wine-red colored liquid.

2. ^{1}H NMR and ^{13}C NMR spectra of M-BBO

^{1}H NMR and ^{13}C NMR were measured by Bruker Advance 400 NMR spectrometer at the resonant frequency of 400 MHz with $CDCl_3$ as the solvent. Magna-IR 560 infrared spectrometer was used with a resolution of 2 cm^{-1} in the range of 4000-500 wave numbers.

M-BBO: ^{1}H NMR (400 MHz, $CDCl_3$): δ=8.91 (d, *J* = 7.3 Hz, 1H), 8.72 (d, *J* = 7.3 Hz, 1H), 8.25 (d, *J* = 8.1 Hz, 1H), 8.14 (d, *J* = 8.2 Hz, 1H), 7.78 (dd, *J* = 16.5, 8.2 Hz, 2H), 7.50 (dd, *J* = 22.0, 8.4 Hz, 2H). ^{13}C NMR (400 MHz, $CDCl_3$): δ = 159.51, 151.59, 145.27, 135.15, 132.69, 132.39, 132.19, 132.13, 132.01, 129.85, 128.33, 127.42, 127.15, 127.12, 123.82, 120.31, 113.36, 106.47.

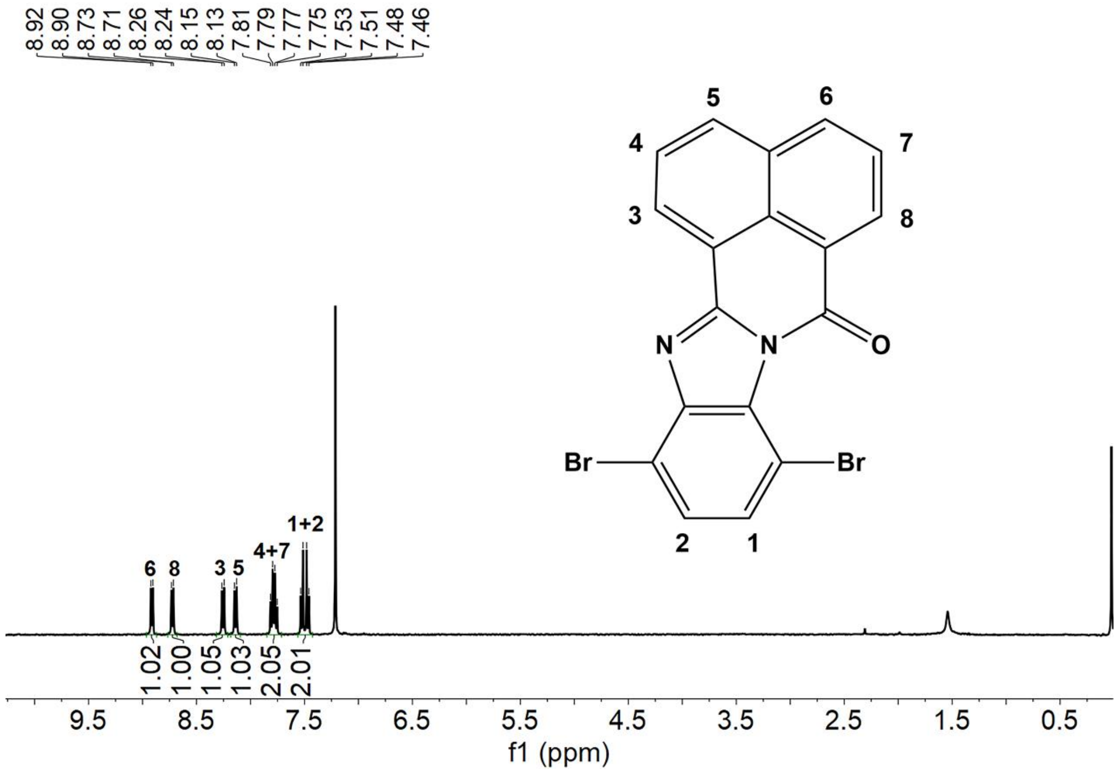

Figure S1. The ^{1}H NMR spectrum of M-BBO.

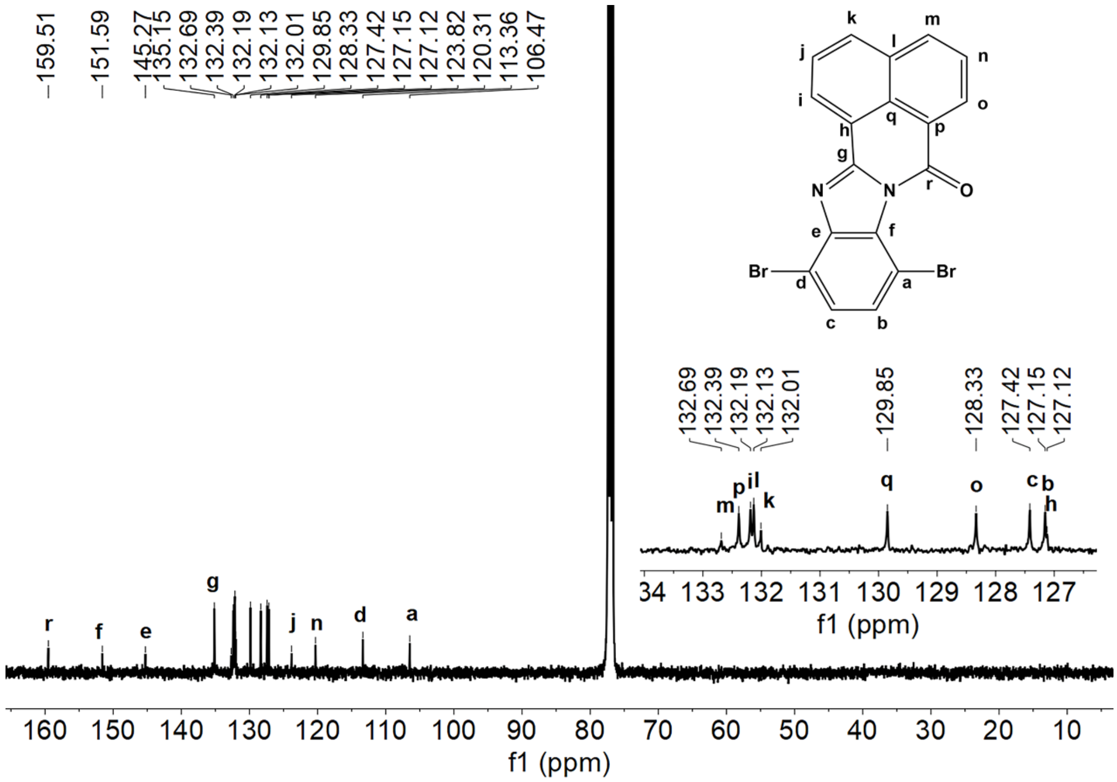

Figure S2. The ^{13}C NMR spectrum of M-BBO.

3. ^{1}H NMR spectrum of PF-BBO

PF-BBO: ^{1}H NMR (400 MHz, $CDCl_3$): δ=9.13-7.25 (m, 14H, Ar-H), 2.44-1.77 (m, 4H, CH_2), 1.14 (dd, J = 33.8 Hz, 24H, CH_2), 0.78 (s, 6H, CH_3). In the chemical shift range of 7.25 to 9.13 are aromatic hydrogens, and in the chemical shift range of 0.78 to 2.44 are hydrogens in alkyl chains.

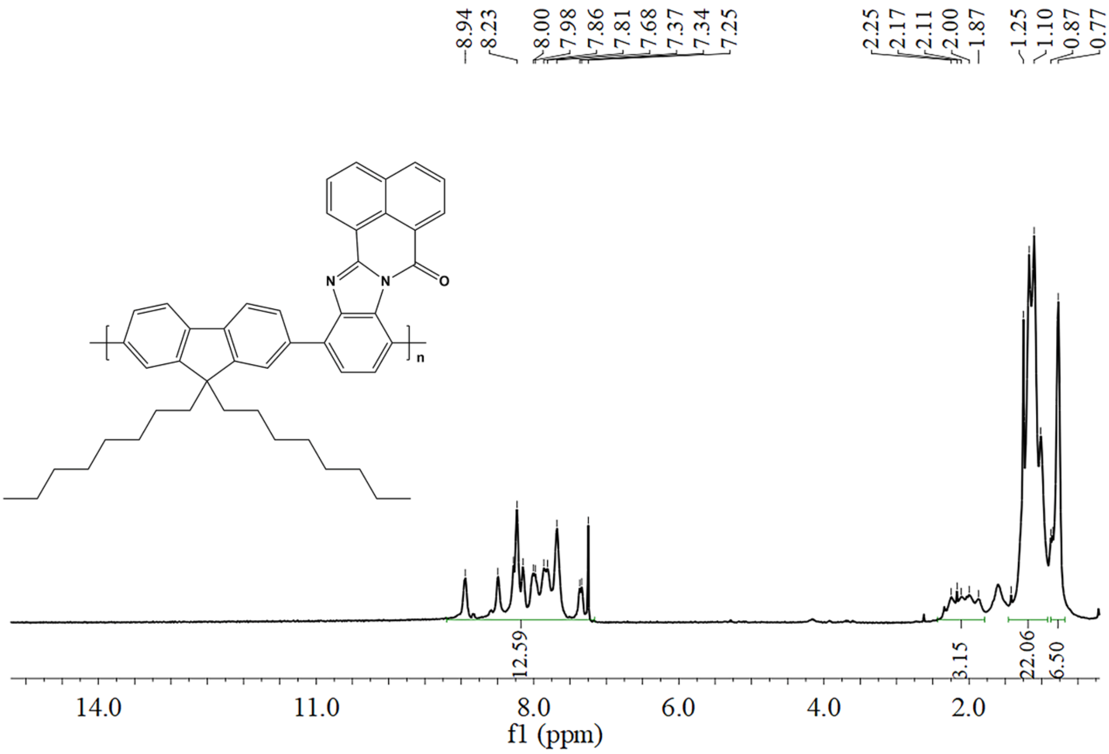

Figure S3. The ^{1}H NMR spectrum of PF-BBO.

4. FT-IR spectrum of PF-BBO

Fourier transform infrared (FT-IR) spectra were indicated on Magna-IR560 infrared spectrometers with KBr pellet in the range of 4000-500 wavenumbers and at a resolution of 2 cm^{-1}. The FT-IR spectra of PF-BBO, clearly indicating the characteristic aromatic C−H stretching vibration absorption peak at 2925 cm^{-1}, with aliphatic C−H

stretching at vibration absorption peak 2850 cm^{-1}, C=O stretching at vibration absorption peak 1717 cm^{-1}, C=C stretching vibration absorption peak at 1616-1483 cm^{-1}, and C−N stretching vibration absorption peak at 1238 cm^{-1}.

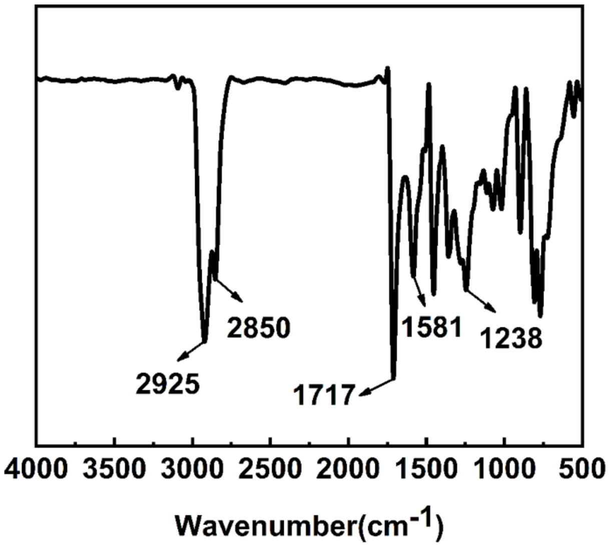

Figure S4. The FT-IR spectrum of PF-BBO.

5. Molecular weight and dispersion of PF-BBO

Table S1. Molecular weight and dispersion of PF-BBO.

	$\overline{Mn}$ (Da) [a]	$\overline{Mw}$ (Da) [b]	PDI ($\overline{Mw}/\overline{Mn}$) [c]
PF-BBO	28802	29586	1.03

[a] Number-average molecular weight

[b] Weight-average molecular weight

[c] Polydispersity index

6. Optical and electrochemical properties

The PF-BBO exhibit two broad absorption bands in the range of

220-270 nm and 290-380 nm in both THF solution and film conditions. The high wavelength absorption band should be attributed to the inherent intramolecular charge transfer transition of the D-A molecules, and the π−π* transition of the conjugated skeleton is the main reason leading to the low wavelength absorption band. Relative to the solution, the absorption maximum of film red shifts in virtue of the strong interchain interaction between the polymer backbone and the intermolecular π-stacking, which is favorable for rapid charge transport (Figure S5a, b). The optical band gap value of the PF-BBO (E_g^{opt}) can be calculated by the absorption edge wavelength (λ_{onset}) of the film absorption spectrum. Figure S5c displays the maximum emission peak of the PF-BBO fluorescence spectrum. To evaluate the electrochemical properties of the PF-BBO, the cyclic voltammetry (CV) measurements were measured in anhydrous CH_3CN with 0.1 M Bu_4NClO_4 as the electrolyte, as shown in Figure S5d. The highest occupied molecular orbital (HOMO) and the lowest unoccupied molecular orbital (LUMO) were calculated using the initial oxidation potential (E_{onset}) and the optical band gap value (E_g^{opt}), respectively (Table S2). In comparison to HOMO and ITO electrodes, the potential barrier between the Al electrode and the LUMO energy level is substantially larger. As a result, it is simpler to inject electrons into HOMO holes

from the ITO electrode. Therefore, the hole injection dominates the conduction process.

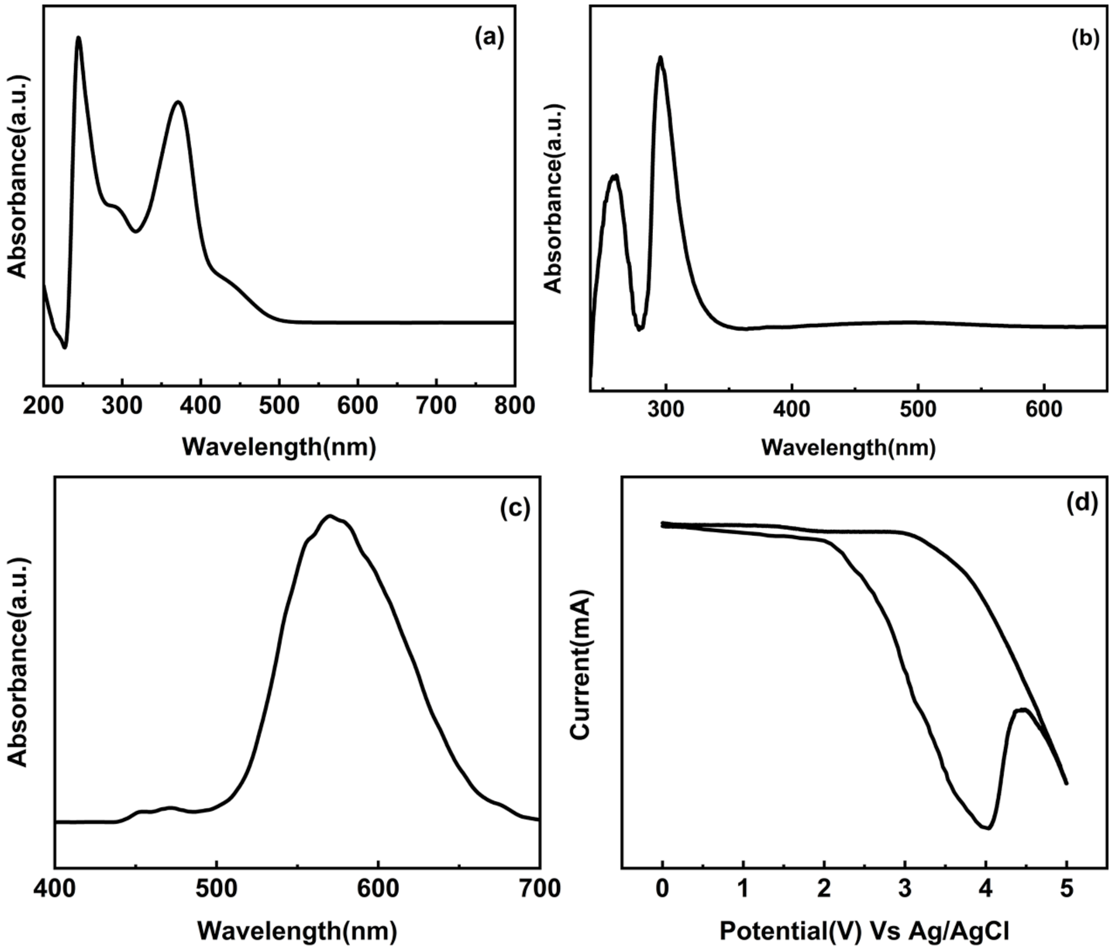

Figure S5. (a) UV/Vis spectrum of PF-BBO film; (b) UV/Vis spectrum of PF-BBO solution; (c) PL spectrum of PF-BBO; (d) CV curve of PF-BBO.

Table S2. Photoelectric properties of PF-BBO.

	[a] λ_{onset} (nm)	PL_{max} (nm)	[b] E_g^{opt} (eV)	[c] E_{onset} (V)	[d] E_{HOMO}	[d] E_{LUMO}
PF-BBO	412	585	3.00	2.18	-5.59	-2.59

[a] λ_{onset}: Measured in the PF-BBO solution with a concentration of

about 1×10^{5} mol·L^{-1} in THF

[b] $E_g^{opt}=1240/\lambda_{onset}$

[c] E_{onset}: Initial potential of the CV curve

[d] $E_{HOMO}=-(E_{onset} + 4.8 - E_{ferrocene})$ eV.; $E_{LUMO}= E_{HOMO} + E_g^{opt}$
